# Supplementary material for: Interplay of Dietary Fatty Acids and Cholesterol Impacts Brain Mitochondria and Insulin Action
Source: Nutrients. 2020 May 23;12(5):1518. doi: 10.3390/nu12051518 (PMC7284591; doi:10.3390/nu12051518)
Supplement: Supplementary file 1 [file nutrients-12-01518-s001.pdf]

Interplay of Dietary Fatty Acids and Cholesterol Impacts Brain Mitochondria and Insulin Action

Mareike Schell [1,2,†], Chantal Chudoba [1,2,†], Antoine Leboucher [1,2], Eugenia Alfine [1], Tanina Flore [1], Katrin Ritter [1], Katharina Weiper [1,3], Andreas Wernitz [4], Janin Henkel [3,#] and André Kleinridders [1,2,5,#,*]

SUPPLEMENTARY MATERIAL

***In vivo* measurements**

Oral glucose tolerance test (GTT) was performed in week 18 of the feeding intervention after 16 hours of fasting by oral gavage of glucose (2 mg/kg body weight). Insulin tolerance (ITT) test was performed in week 19 of the feeding intervention after 4 h of fasting by intraperitoneal injection of insulin (0.25 IU/kg body weight). Blood glucose from tail vein was monitored at 0 min, 15 min, 30 min, 60 min, 90 min and 120 min using a glucose sensor (Breeze2, Bayer; Berlin, Germany).

MTT assay

To assess a non-toxic concentration of MβCD and cholesterol for CLU183 cells, MTT assay was performed. For this, cells were stimulated with various concentrations of either MβCD or cholesterol for 16 h and following incubated with 0.5 mg/ml of tetrazolium dye 3-(4,5-dimethylthiazol-2-yl)-2,5-diphenyltetrazolium bromide (MTT; Sigma-Aldrich, Taufkirchen, Germany) for 3 h. Sodium-dodecyl sulfate (SDS; Carl Roth, Karlsruhe, Germany) was used as positive control to induce cell death. Afterwards, dimethyl sulfoxide (DMSO; Sigma-Aldrich, Taufkirchen, Germany) was used to solubilize formazan which is the reduced form of MTT. Optical density was determined at 560 nm and 670 nm (reference wavelength) using a plate reader (TECAN, Männedorf, Switzerland).

Supplementary Table S1. Diet composition.

	STD	CHO+STD	CHO+SOY	LAR	CHO+LAR
Metabolizing energy (kcal/g)	3.06	3.06	4.64	4.73	4.73
Energy from carbohydrates (%)	65	65	35	35	35
Energy from protein (%)	25	25	16	20	20
Energy from fat (%)	10	10	49	45	45
Cholesterol (%)	0.00	0.75	0.75	0.00	0.75
Fatty acid composition					
Saturated fatty acids (g/100g)	0.55	0.55	4.00	7.26	7.26
Mono-unsaturated fatty acids (g/100g)	0.64	0.64	5.75	8.69	8.69
Poly-unsaturated fatty acids (g/100g)	2.01	2.01	14.50	6.36	6.36

Diet composition. Mice diets used in the feeding experiment. Standard chow diet (STD), 0.75% cholesterol in a standard diet (CHO+STD), 0.75% cholesterol in a high fat diet containing ω6-PUFA-rich soybean oil (CHO+SOY), high fat diet containing mainly lard as fat source (LAR), 0.75% cholesterol in a high fat diet containing mainly lard as fat source (CHO+LAR).

Supplementary Table S2. Primer pairs specific for each gene used in the study.

Name	Fw Primer Sequence (5´- 3´)	Rv Primer Sequence (5´- 3´)
**Hmgcr**	AGTACATTCTGGGTATTGCTGG	ACTCGCTCTAGAAAGGTCAATC
**Srebp2**	GCGTTCTGGAGACCATGGA	ACAAAGTTGCTCTGAAAACAAATCA
**Fdps**	CTGCAGAGTTCCTATCAGACAG	CAGGTAGAAAGAGTAGAAAGCCG
**Sqle**	TTGTTGCGGATGGACTCTTCTCCA	GTTGACCAGAACAAGCTCCGCAAA
**Hsp60**	AGTGTTCAGTCCATTGTCCC	TGACTGCCACAACCTGAAG
**Sirt3**	CGGCTCTATACACAGAACATCG	CATCAGCCCATATGTCTTCCC
**Pgc1α**	AGCCGTGACCACTGACAACGAG	GCTGCATGGTTCTGAGTGCTAAG
**Mfn1**	CATTGCGTTTCGGTTTTCCC	GAAGGAGCAGTAGGAGTTGAAG
**Drp1**	TCCCAATTCCATTATCCTCGC	CATCAGTACCCGCATCCATG
**Opa1**	GTGTGCTGGAAATGATTGCTC	TGGTGAGATCAAATTCCCGAG
**Cox2**	CCTGGTGAACTACGACTGCT	GAATAACCCTGGTCGGTTTG
**Cox3**	GCAGGATTCTTCTGAGCGTTCT	GTCAGCAGCCTCCTAGATCATGT
**Nd1**	GGATCCGAGCATCTTATCCA	GGTGGTACTCCCGCTGTAAA
**Nd6**	ATTAAACAACCAACAAACCCAC	TTTGGTTGGTTGTCTTGGGTT
**Tbp**	CTGGAATTGTACCGCAGCTT	ATGATGACTGCAGCAAATCG
**β-Actin**	GCCAACCGTGAAAAGATGAC	TACGACCAGAGGCATACAG

Supplementary Figure S1: High-fat diets decrease insulin sensitivity.

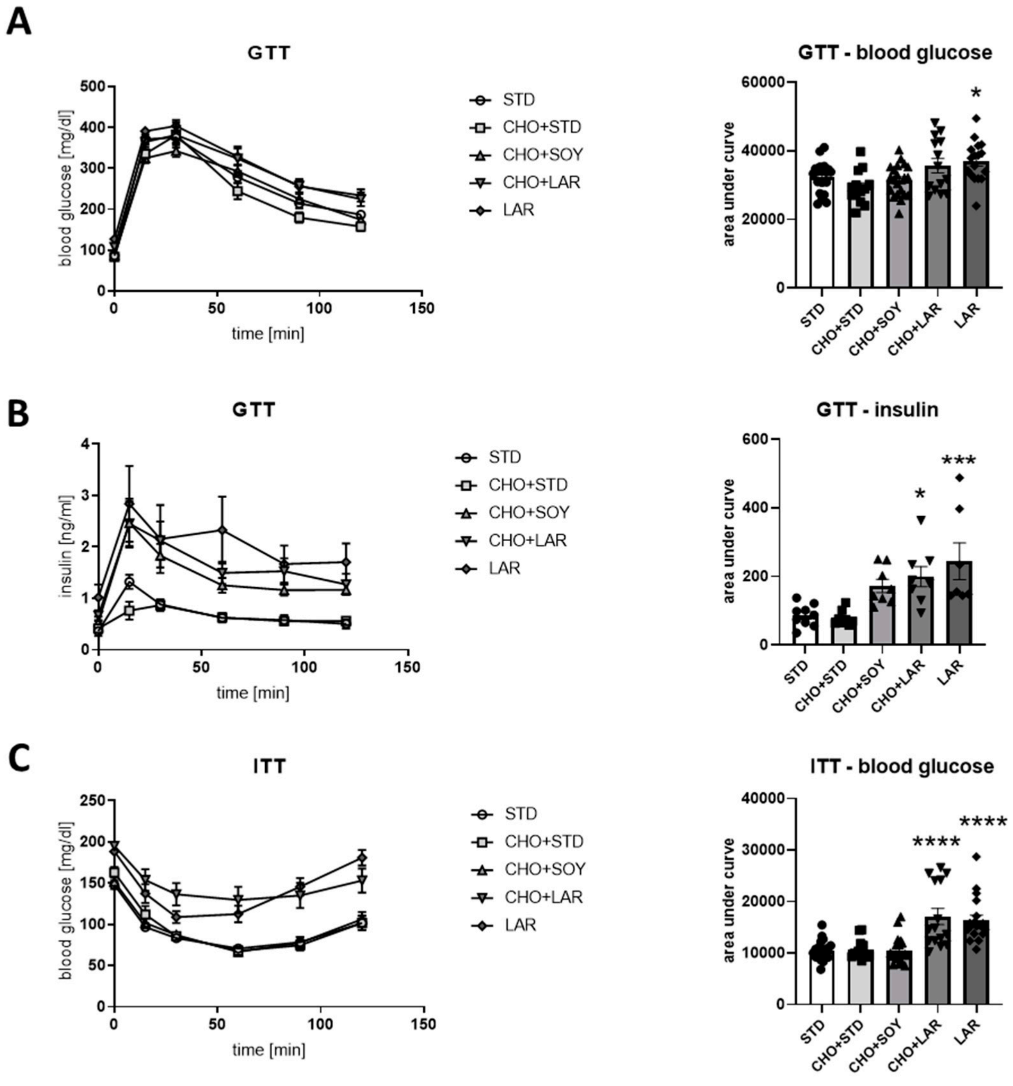

Supplementary Figure S1: High-fat diets decrease insulin sensitivity. (**A,B**) Glucose tolerance test (GTT) and (**C**) insulin tolerance test (ITT) after 18 and 19 weeks of feeding intervention. All values are displayed as median ± SEM with a total n of 13-21 per group. Statistics: One-way-ANOVA with Dunnett post hoc test for multiple comparisons. * $p < 0.05$, *** $p < 0.001$, **** $p < 0.0001$. *: vs STD

Supplementary Figure S2: Changes in relative abundance of fatty acids in serum due to dietary intervention.

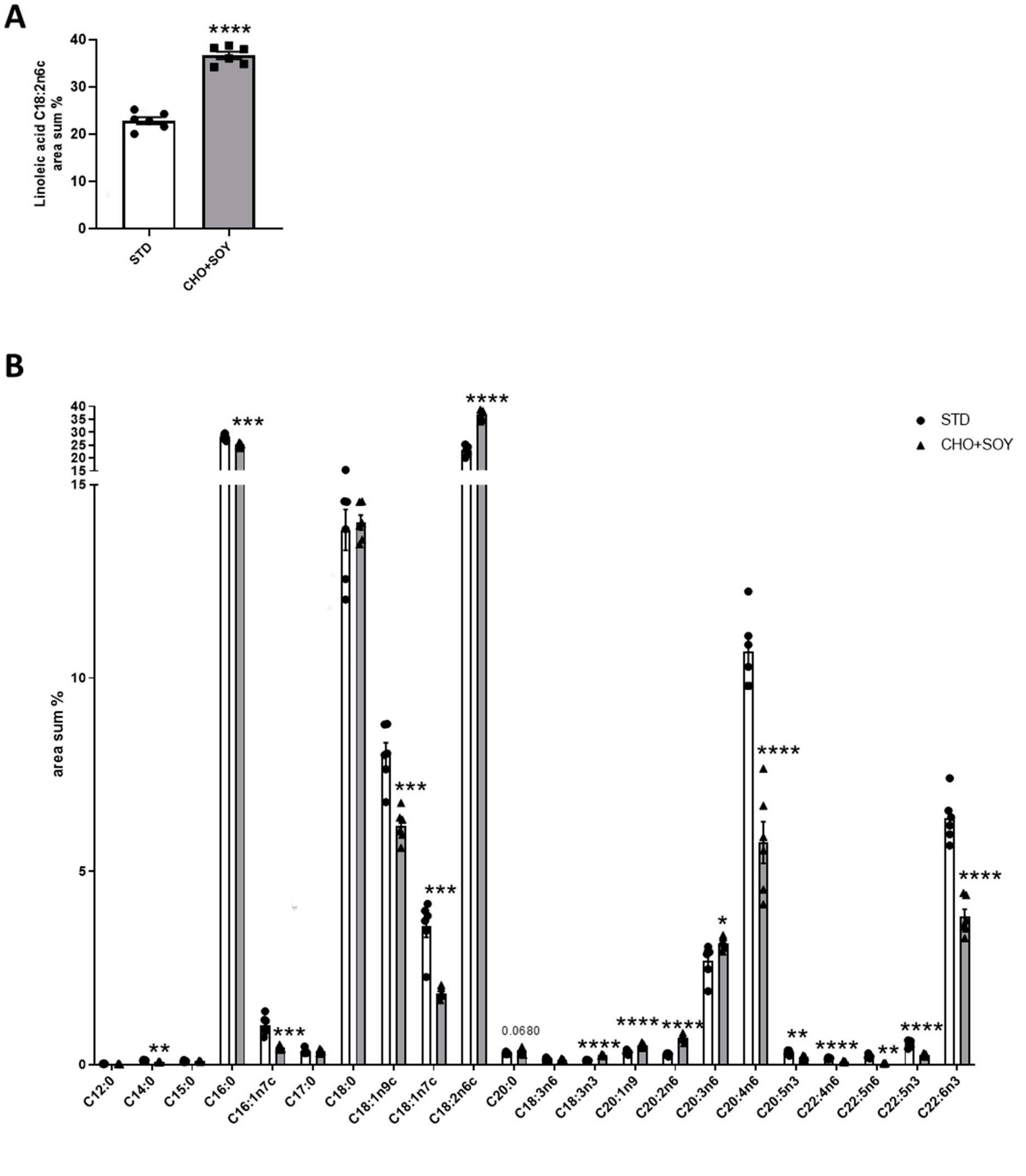

Supplementary Figure S2. Changes in relative abundance of fatty acids in serum due to dietary intervention. (**A**) Relative abundance of linoleic acid in serum. (**B**) Relative fatty acid composition in serum**.** All values are displayed as median ± SEM with a total n of 6 mice per group. Statistics: Multiple Student's t-test for unpaired samples. * $p < 0.05$, ** $p < 0.01$, *** $p < 0.001$, **** $p < 0.0001$ vs STD group.

Supplementary Figure S3: Determination of concentration for MβCD and cholesterol in hypothalamic neurons.

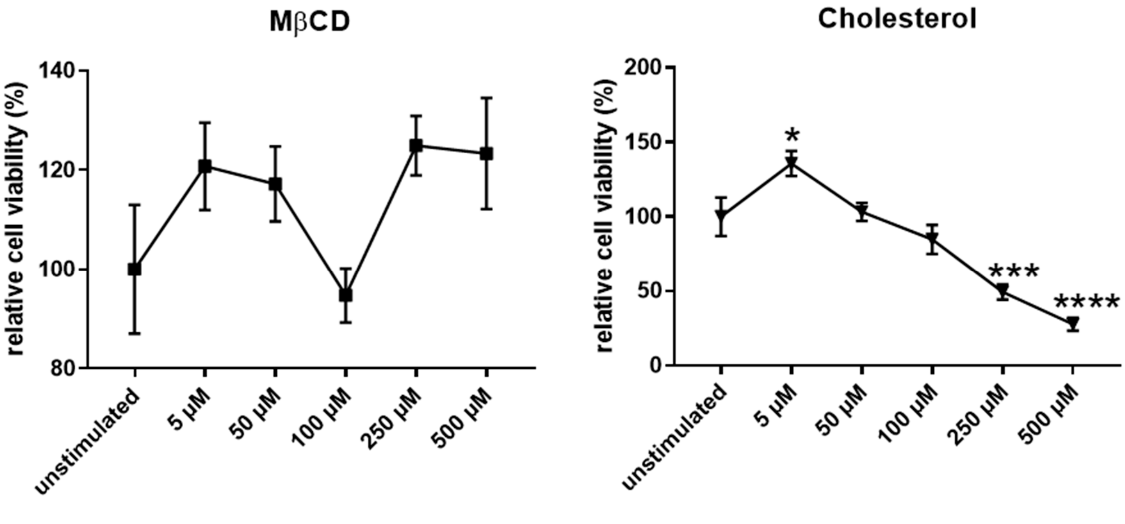

Supplementary Figure S3. Determination of concentration for MβCD and cholesterol in hypothalamic neurons. Cell viability in % relative to unstimulated CLU183 cells using MTT assay with a total n of 8 per group. Statistics: One-way-ANOVA with Dunnett's multiple comparisons test. All the data are presented as mean ± SEM. * $p < 0.05$, *** $p < 0.001$, **** $p < 0.0001$ vs unstimulated control.

Supplementary Figure S4: Cholesterol regulates endogenous cholesterol biosynthesis in hypothalamic neurons.

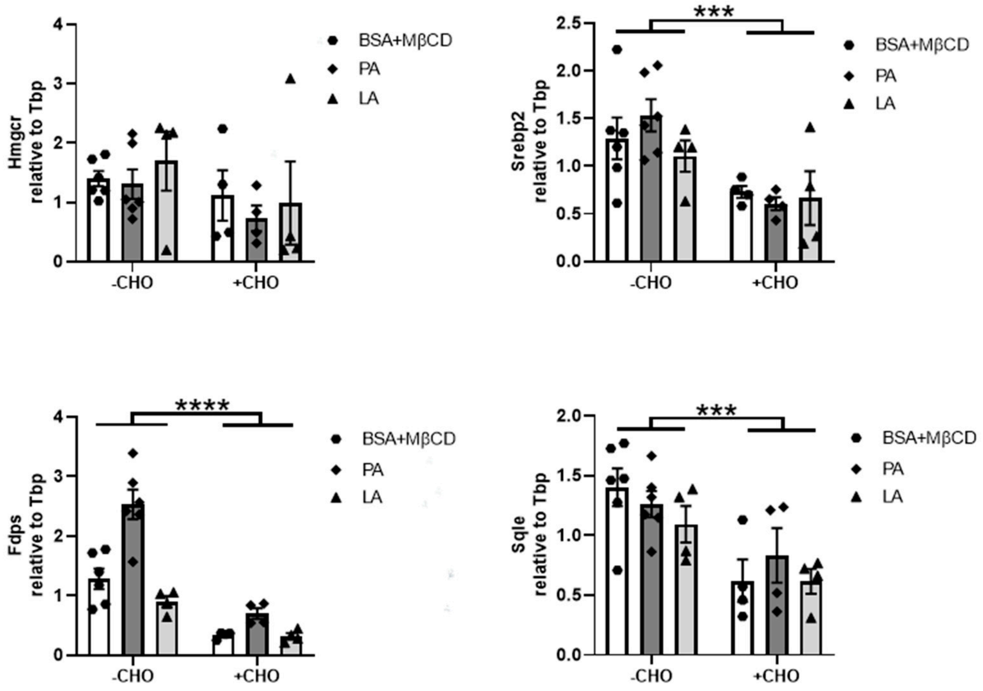

Supplementary Figure S4: Cholesterol regulates endogenous cholesterol biosynthesis in hypothalamic neurons. Relative mRNA expression of *Hmgcr*, *Srebp2*, *Fdps*, and *Sqle*. Data of three independent experiments with a total n= 4-6. All the data are presented as mean ± SEM. *** $p < 0.001$, **** $p < 0.0001$.

Supplementary Figure S5: Cholesterol regulates mitochondrial function and dynamics in hypothalamic neurons.

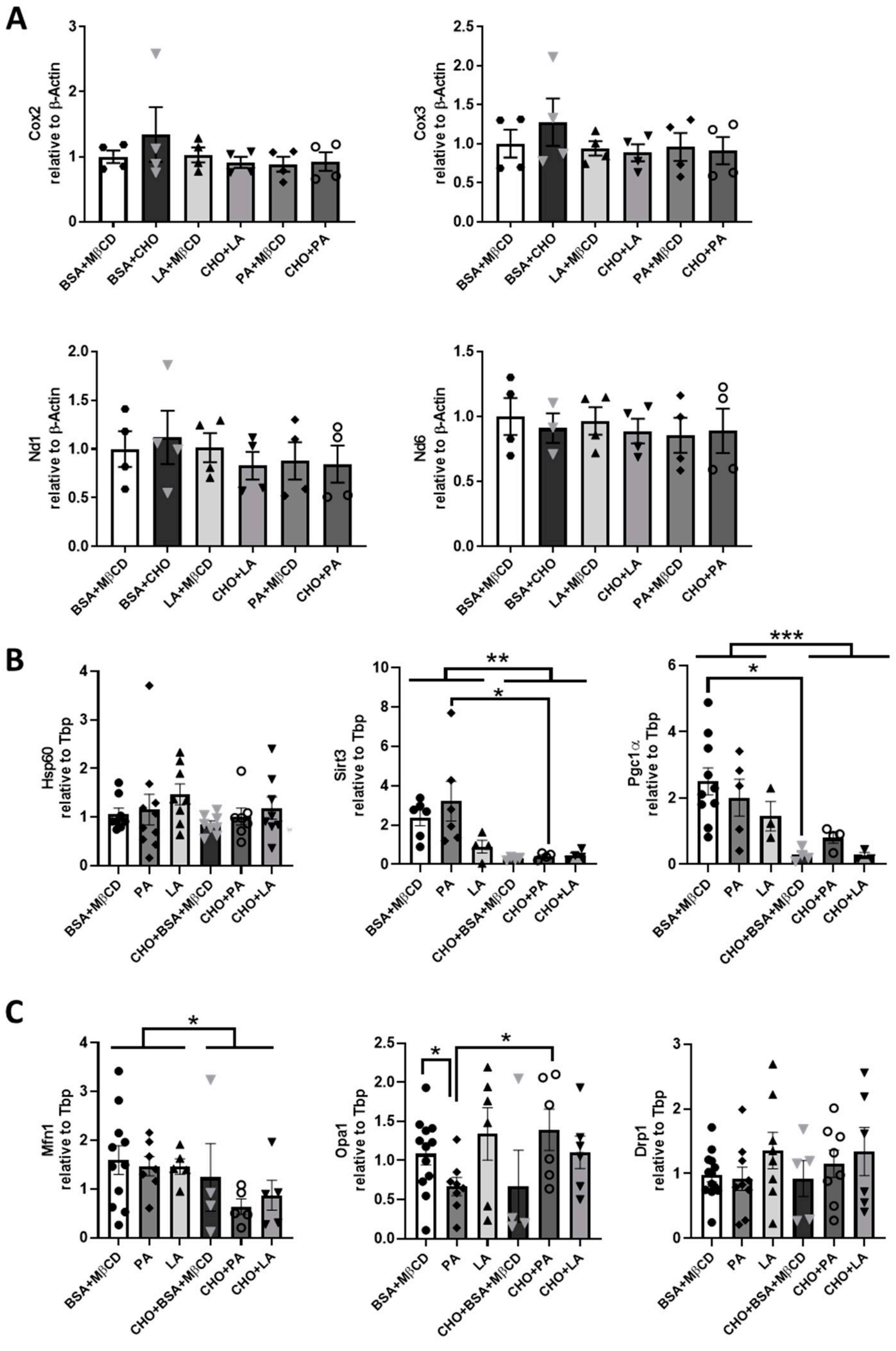

Supplementary Figure S5. Cholesterol regulates mitochondrial function and dynamics in hypothalamic neurons. (**A**) Relative mRNA expression of *Cox2*, *Cox3*, *Nd1*, and *Nd6* in stimulated neurons. Data of four independent experiments with a total n= 4. Relative mRNA expression of (**B**) *Hsp60*, *Sirt3*, *Pgc1α*, (**C**) *Mfn1*, *Opa1*, and *Drp1* in stimulated neurons. Data of three independent experiments with a total n= 4-12. All the data are presented as mean ± SEM. * $p < 0.05$, ** $p < 0.01$, *** $p < 0.001$.

Supplementary Figure S6: Characterization of insulin receptor-deficient hypothalamic neurons.

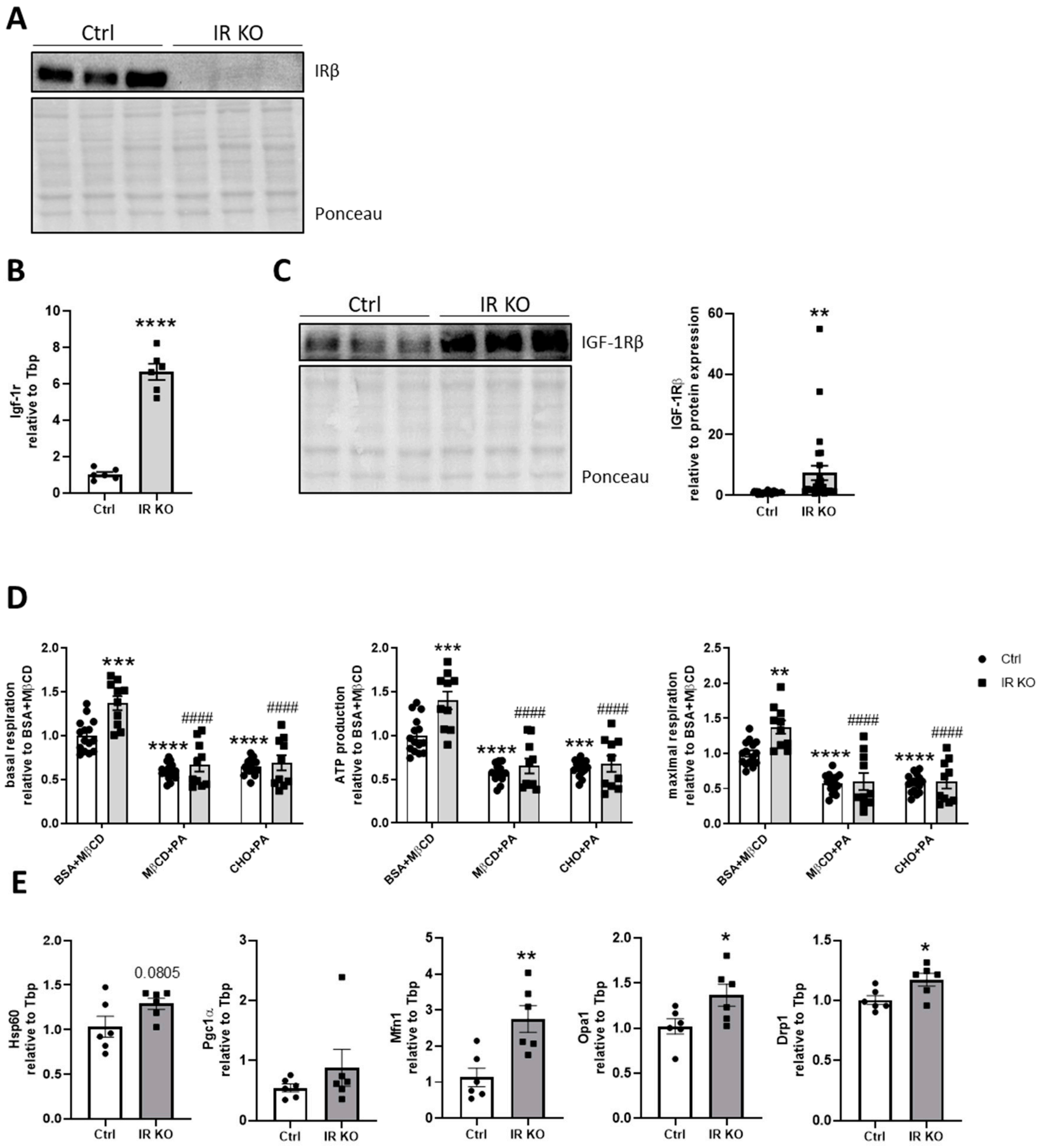

Supplementary Figure S6. Characterization of insulin receptor-deficient hypothalamic neurons. (**A**) Protein expression of IR in control and IR KO CLU183 cells. (**B**) Relative mRNA expression of Igf-1r. (**C**) Protein expression of IGF-1R in control and IR KO CLU183 cells and densitometric analysis. (**D**) Relative oxygen consumption rate (basal respiration, ATP production, and maximal respiration) in stimulated control and IR KO cells. (**E**) Relative mRNA expression of *Hsp60*, *Pgc1α*, *Mfn1*, *Opa1*, and *Drp1* in control and IR KO cells. (B) Dense intensity of IGF-1R was normalized to Ponceau, which was verified on the same Western blot membrane as a loading control and calculated relative to the control (Ctrl) group. A representative blot is shown. (B-E) Data of three independent experiments with a total n= 6-26. All the data are presented as mean ± SEM. Statistics: Multiple Student's t-test for unpaired samples and one-way-ANOVA with Tukey's post hoc test for multiple comparisons., * $p < 0.05$, ** $p < 0.01$, *** $p < 0.001$, **** $p < 0.0001$. * vs Ctrl: BSA+MβCD, #### vs IR KO: BSA+MβCD.

Supplementary Figure S7: Palmitate but not cholesterol induces JNK activation.

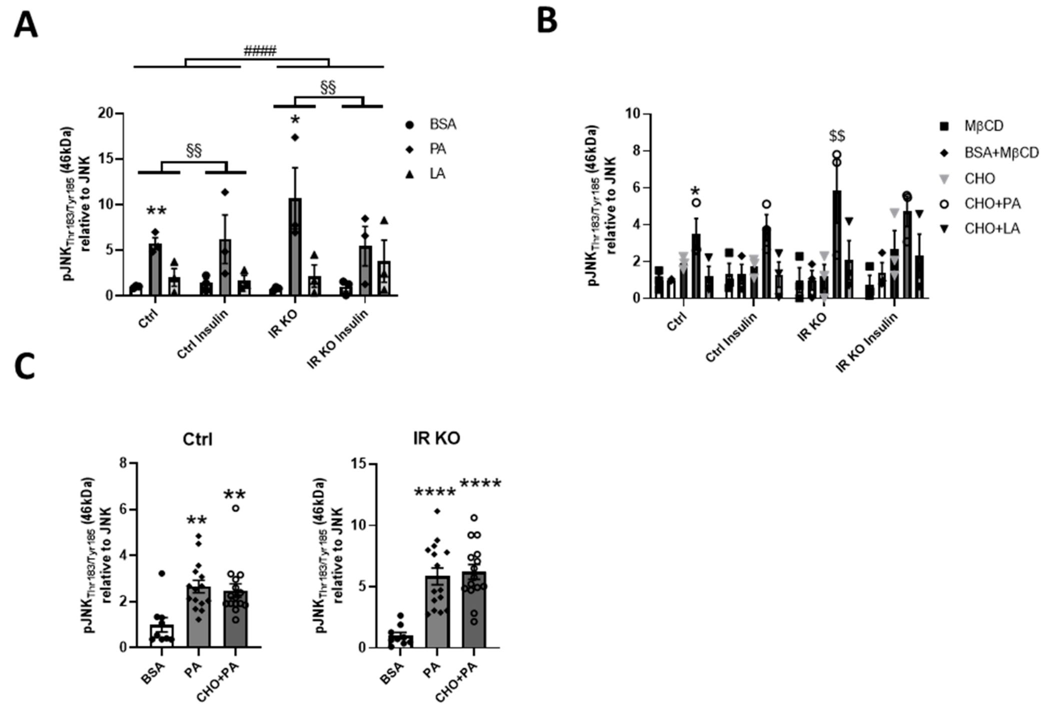

Supplementary Figure S7. Palmitate but not cholesterol induces JNK activation. (**A-C**) Densitometric analysis of the protein expression of pJNK Thr183/Tyr185 and JNK in stimulated control or IR KO CLU183 cells. All values are displayed as median ± SEM. Data of three independent experiments with a total n= 3-15. Dense intensity of pJNK Thr183/Tyr185 to total JNK protein, which was verified on the same Western blot membrane as a loading control and calculated relative to the respective control (BSA or BSA+MβCD) group in each gel. Statistics: Two-way-ANOVA with Tukey's post hoc test for multiple comparisons of Ctrl vs IR KO and one-way-ANOVA with Tukey's post hoc test for multiple comparisons. * $p < 0.05$, ** $p < 0.01$, **** $p < 0.0001$. *: vs BSA(Ctrl) or BSA+MβCD(Ctrl), $: vs BSA(IRKO) or BSA+MβCD(IRKO), §§: unstimulated vs Insulin stimulated, ####: Ctrl vs IR KO.

Supplementary Figure S8: Investigating different control stimulations on insulin sensitivity in hypothalamic neurons.

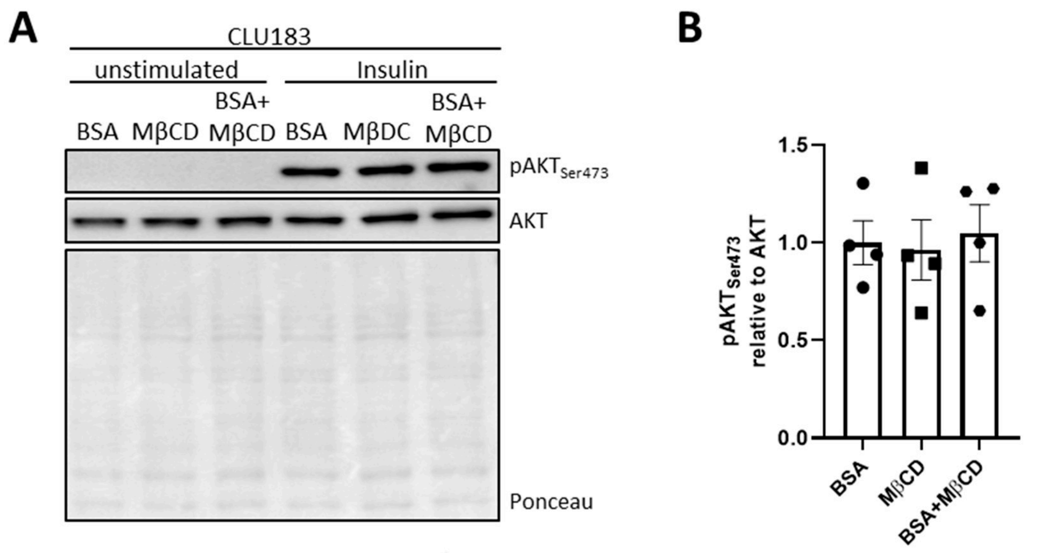

Supplementary Figure S8. Investigating different control stimulations on insulin sensitivity in hypothalamic neurons. (**A**) Protein expression of pAKT Ser473, AKT and (**B**) densitometric analysis in stimulated neurons. Dense intensity of pAKT Ser473 was normalized total AKT, which was verified on the same Western blot membrane as a loading control and calculated relative to the control (BSA) group. A representative blot is shown. All values are displayed as median ± SEM. Data of four independent experiments with a total n = 4. Statistics: One-way-ANOVA with Tukey’s post hoc test for multiple comparisons.

Supplementary Figure S9: Palmitate- and palmitate/cholesterol-induced insulin resistance is independent of JNK activation.

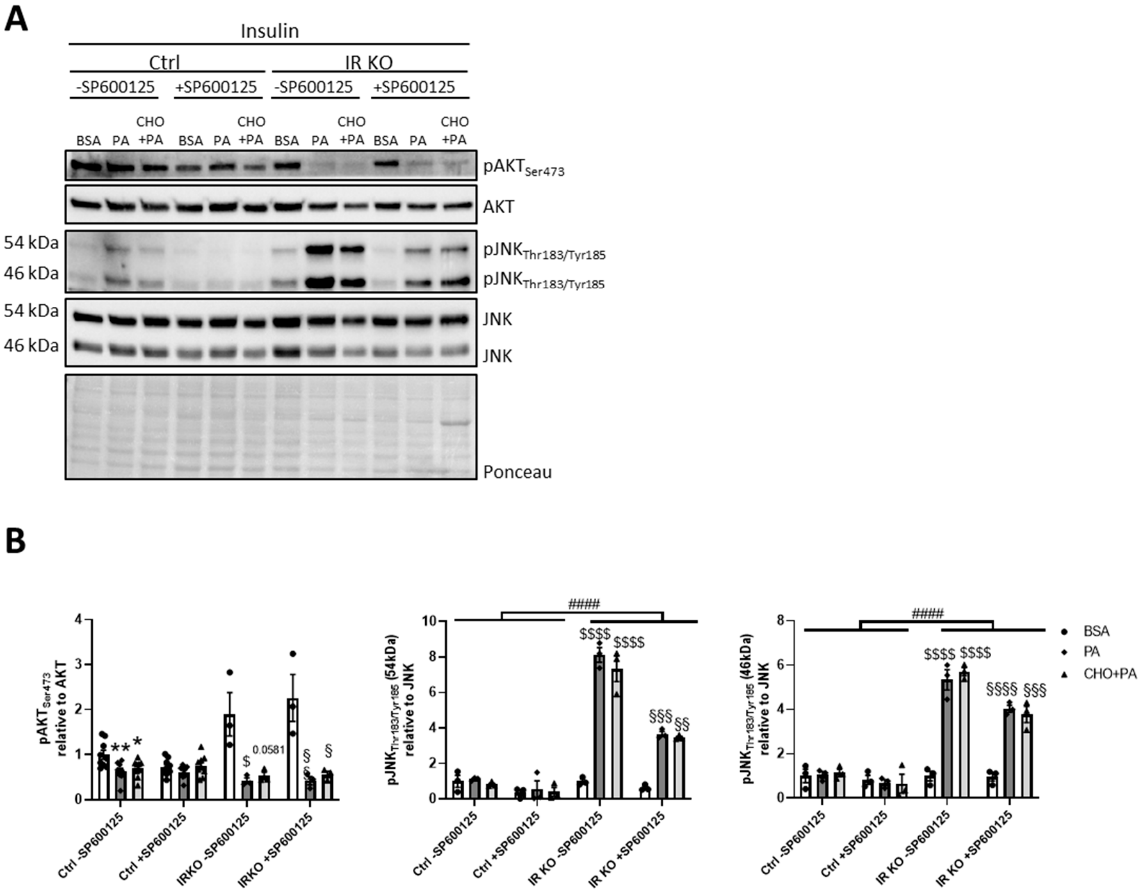

Supplementary Figure S9. Palmitate- and palmitate/cholesterol-induced insulin resistance is independent of JNK activation. (**A**) Protein expression in stimulated control or IR KO CLU183 cells of pAKT Ser473, AKT, pJNK Thr183/Tyr185, JNK and (**B**) densitometric analysis. Dense intensity of pAKT Ser473 was normalized total AKT protein and for pJNK Thr183/Tyr185 to total JNK protein, which was verified on the same Western blot membrane as a loading control and calculated relative to the control (BSA) group in each gel. A representative blot is shown. All values are displayed as median ± SEM. Data of three independent experiments with a total n = 3-9. Statistics Two-way-ANOVA with Tukey's post hoc test for multiple comparisons of Ctrl vs IR KO and one-way-ANOVA with Tukey's post hoc test for multiple comparisons * $p < 0.05$, ** $p < 0.01$, *** $p < 0.001$, **** $p < 0.0001$. *: vs Ctrl -SP600125 BSA, $, $$$$: vs IR KO -SP600125 BSA, §, §§, §§§, §§§§: vs IR KO +SP600125 BSA, #: Ctrl vs IR KO, 0.0581 vs IR KO -SP600125.
